# Comparison of Radiological and Clinical Outcomes in Patients Treated with Standard Plating versus Intramedullary Nailing in Distal Tibial Fracture

**DOI:** 10.1111/os.13210

**Published:** 2022-02-01

**Authors:** Dong‐Il Chun, Tae‐Hong Min, Eun Myeong Kang, Woojin Yu, Sung Hun Won, Jaeho Cho, Young Yi

**Affiliations:** ^1^ Department of Orthopaedic Surgery Soonchunhyang University College of Medicine Seoul South Korea; ^2^ Department of Orthopaedic Surgery College of Medicine, University of Ulsan, Asan Medical Center Seoul South Korea; ^3^ Department of Orthopaedic Surgery Chuncheon Sacred Heart Hospital, Hallym University Chuncheon‐si South Korea; ^4^ Department of Orthopaedic Surgery Seoul Foot and Ankle Center, Inje University Seoul South Korea

**Keywords:** Bone nails, Bone plates, Fracture fixation, Internal/methods, Intramedullary/methods, Tibial fractures/surgery

## Abstract

**Objective:**

To evaluate clinical and radiological outcomes including hindfoot alignment after plate *vs* intramedullary nailing (IMN) for distal tibia fracture and to define radiologic parameters that influence changes in hindfoot alignment.

**Methods:**

Among 92 patients with distal tibia metaphyseal fractures treated from 2002 to 2015, 39 cases of intramedullary nailing and 53 cases of standard plate osteosynthesis were performed. Union rate and complication rate were compared in both groups. Radiographic measurements including hindfoot angulation, moment arm, calcaneal pitch angle, and Meary angle were evaluated at a minimum of 1‐year follow‐up. Hindfoot alignment changes after surgery were compared between both groups using student *t*‐test. Correlation and regression were analyzed between fracture alignment parameters and hindfoot alignment.

**Results:**

All patients ultimately healed, with an average union period of 26 weeks in both groups. The AOFAS and VAS scores were not significantly different between the two groups. Complications were similar between the two groups. Hindfoot alignment angle, calcaneal pitch, and Meary angle showed no significant differences between the groups. The hindfoot moment arm increased with valgus in the IMN group. A low correlation was detected between angulation at the fracture site in the coronal view and hindfoot alignment (angulation and moment arm) changes (*R* = 0.38). A significantly high correlation was noted only between transverse rotation and hindfoot alignment changes (*R* = 0.79).

**Conclusions:**

Rotation in the transverse plane notably influenced changes in hindfoot alignment. And this suggests that patients with distal tibia fracture should be closely monitored for hindfoot alignment changes caused by intraoperative transverse rotation regardless of the fixation method.

## Graphical abstract

Reduction and stable fixation of distal extra‐articular tibia fractures with intramedullary nail is often technically challenging because of a large medullary cavity within a short distal fragment. This article evaluates clinical and radiological outcomes including hindfoot alignment after plate *vs* intramedullary nail for distal tibia fracture and to define radiologic parameters that influence changes in hindfoot alignment. In this study, 92 patients with distal tibia metaphyseal fractures treated with intramedullary nailing and standard plate osteosynthesis from 2002 to 2013 were included. Clinical and radiologic comparison were made between standard plate and intramedullary nail group. Relationship of ankle alignment changes and hindfoot alignment changes were evaluated. The result showed angulation on coronal plane after internal fixation may slightly affect hindfoot alignment due to compensation on the subtalar joint. However, rotation on the transverse plane has intimately influenced hindfoot alignment.

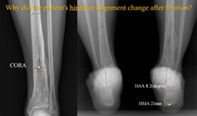

## Introduction

Fractures of the distal tibia occur commonly, affecting patients of all ages. On one end of the spectrum, low‐energy falls generate torsional spiral fractures of the metaphysis and distal diaphysis, whereas, on the other end, high‐energy blunt impacts cause complex comminuted fractures. Even with the least severe fractures, the overlying soft‐tissue envelope is problematic because of the subcutaneously placed anteromedial cortex of the tibia^1^. In other words, the injury spectrum on distal tibia is so diverse that no single procedure could be applied to all tibia fractures^2^. Hence, the management of distal third tibia fractures is still controversial, and multiple modes of fixation have been discussed in the literature^3^, ^4^, ^5^.

In general, displaced distal tibia shaft fractures are effectively treated with plates and intramedullary nailing (IMN). IMN has been confirmed as a viable alternative to plate osteosynthesis in the management of distal tibia fractures^6^. Additionally, studies that compared the postoperative outcomes of percutaneous locking plate and IMN demonstrated that IMN is more advantageous in reducing the need for secondary procedures, including debridement, hardware removal, and bone grafts^7^. Moreover, a recent meta‐analysis suggested that IMN may be preferable to plates for the fixation of distal tibia metaphyseal fracture, with a lower incidence of infection^8^.

Apart from the advantages of IMN, considering its less invasive nature and potential for earlier weight bearing^9^, it has been associated with malalignment rates as high as 29% and anterior knee pain incidence of 37.5% when used for distal tibia shaft fractures^6^, ^10^, ^11^, ^12^, ^13^. On the other hand, accurate alignment under direct visualization is possible in minimally invasive plate osteosynthesis. Although further exposure of the fracture site could be obtained through a conventional plate osteosynthetic approach, it may result in tissue devitalization, creating an environment that may aggravate the risk of wound complications and implant prominence^10^, ^14^.

Consequently, plate fixation and IMN have been compared in terms of the fracture union, changes in alignment after surgery, change in clinical symptoms, and complications^11^, ^15^, ^16^. However, few studies have focused on changes in foot alignment after distal tibia fracture surgery. Furthermore, changes in the hindfoot and its relevant clinical changes have not been established in the literature.

We hypothesized that, because postoperative alteration in ankle alignment of distal tibia fracture may differ depending on the fixation methods of standard plate (SP) or IMN, differences in hindfoot alignment and clinical outcomes could be observed. Accordingly, the purpose of this study can be categorized into three aspects. First, to compare the clinical outcome depending on the fixation methods of standard plate (SP) or IMN. Second, to compare the radiologic outcome between SP group and IMN group. Third, to investigate the relationship between ankle alignment changes and hindfoot alignment changes.

## Material and Methods

### Inclusion and Exclusion Criteria

In this retrospective study, we enrolled 253 patients with distal tibia metaphyseal fractures (AO‐OTA 43‐A, B, C type) treated by an orthopaedic trauma surgeon from 2002 to 2015. Among them, 161 cases of distal tibia metaphyseal fractures with articular involvement were excluded. Presence of simultaneous ipsilateral fracture, history of previous surgery, severe soft tissue damage including open fracture and existence of a deformity and accompanying tendon or neurovascular injury were also excluded.

### Patient Selection

Overall, 92 patients with distal tibia metaphyseal fractures (AO‐OTA 43‐A1, 43‐A2) with simple or no articular involvement were treated with either SP or IMN. SP group cases were defined as cases in which internal fixation was performed using a locking plate and screw while IMN group cases were defined as cases in which internal fixation was performed using intramedullary nail devices regardless of the reduction method whether through open reduction or a minimally invasive way. There were 53 patients in the SP group and 39 in the IMN group who met the inclusion criteria. No factors influenced the choice of surgical procedure.

This study was approved by the institutional review board of Inje University (PAIK 2019‐05‐003).

### Clinical Measurement

Clinical results were evaluated at a minimum of 1‐year follow‐up. VAS score and limb‐specific outcomes (AOFAS ankle‐hindfoot instrument) were assessed at the final follow‐up.

### Radiological Measurement

Plain weight‐bearing anteroposterior and lateral radiographs of the ankle were taken at a tube‐film distance of 100 cm, with the X‐ray beam projecting parallel to the tibiotalar joint.

**Fig. 1:**
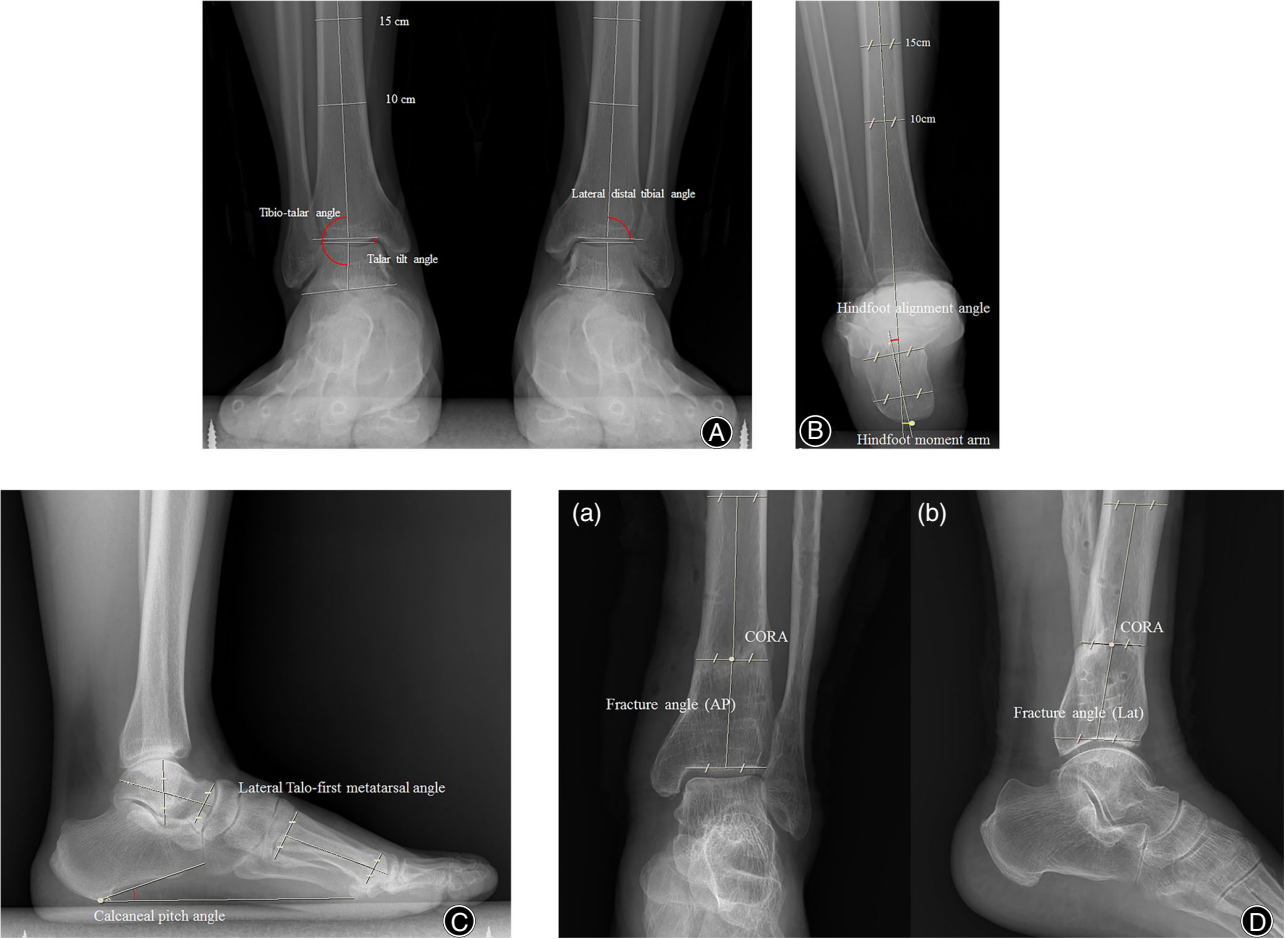
Angular measurements using anteroposterior and lateral weight‐bearing radiographs of the ankle (Maroview version 5.4; Marotech).The images show (A) the lateral distal tibial plafond angle (LDTA, the lateral angle between the longitudinal axis of the tibia and tibial plafond); TTA (tibio‐talar angle, the angle between the tibial axis and talar axis); Talar tilt angle (the angle between talar dome and tibial plafond), (B) HAA (hindfoot alignment angle, the angle between the mid‐diaphyseal axis of calcaneus and the mid‐diaphyseal axis of tibia); HMA (hindfoot moment arm, the distance between the mid‐diaphyseal axis of calcaneus and the mid‐diaphyseal axis of tibia), (C) lateral Mearyʼs angle (angle between the long axis of the talus and first metatarsal bone); Calcaneal pitch angle (the angle between the calcaneal inclination axis and the supporting horizontal surface), (D) fracture angle (the angle between the proximal axis and distal axis of the fracture site)

The hindfoot alignment view was taken with the X‐ray beam projecting 20° from the horizontal plane. We measured the radiological parameters of the lateral distal tibial plafond angle [LDTA], tibiotalar angle, talar tilt angle, tibial rotation angle, hindfoot alignment angle and hindfoot moment arm, calcaneal pitch, and lateral Meary angle (Fig. 1).

The tibial axis was drawn using the same method as described earlier. A line connecting the bisection marks was extended inferiorly and was defined as the tibial axis. The calcaneal axis was drawn by bisecting two pairs of points along the calcaneal cortex. The superior pair of points was made on a line parallel to the subtalar joint at the level just inferior to the sustentaculum tali. The inferior pair of points represent the medial and lateral processes of the calcaneal tuberosity. All measured values were smaller in varus alignment and larger in valgus alignment. The center of rotation of angulation (CORA) is defined as the intersection point between the proximal axis and distal axis of the fracture site. Likewise, fracture angle is the angle between the proximal axis and distal axis of the fracture site.

In order to measure the malalignment of the foot, Meary's angle and a moment arm were investigated as radiographic parameters of midfoot and hindfoot alignment, respectively, and were measured on plain weight‐bearing ankle radiographs in the lateral and hindfoot alignment views, respectively, as described in previous reports. All radiographic parameters were measured accurately using the standard tools of a digital‐based picture archiving and communication system (Maroview version 5.4; Marotech).

**Fig. 2:**
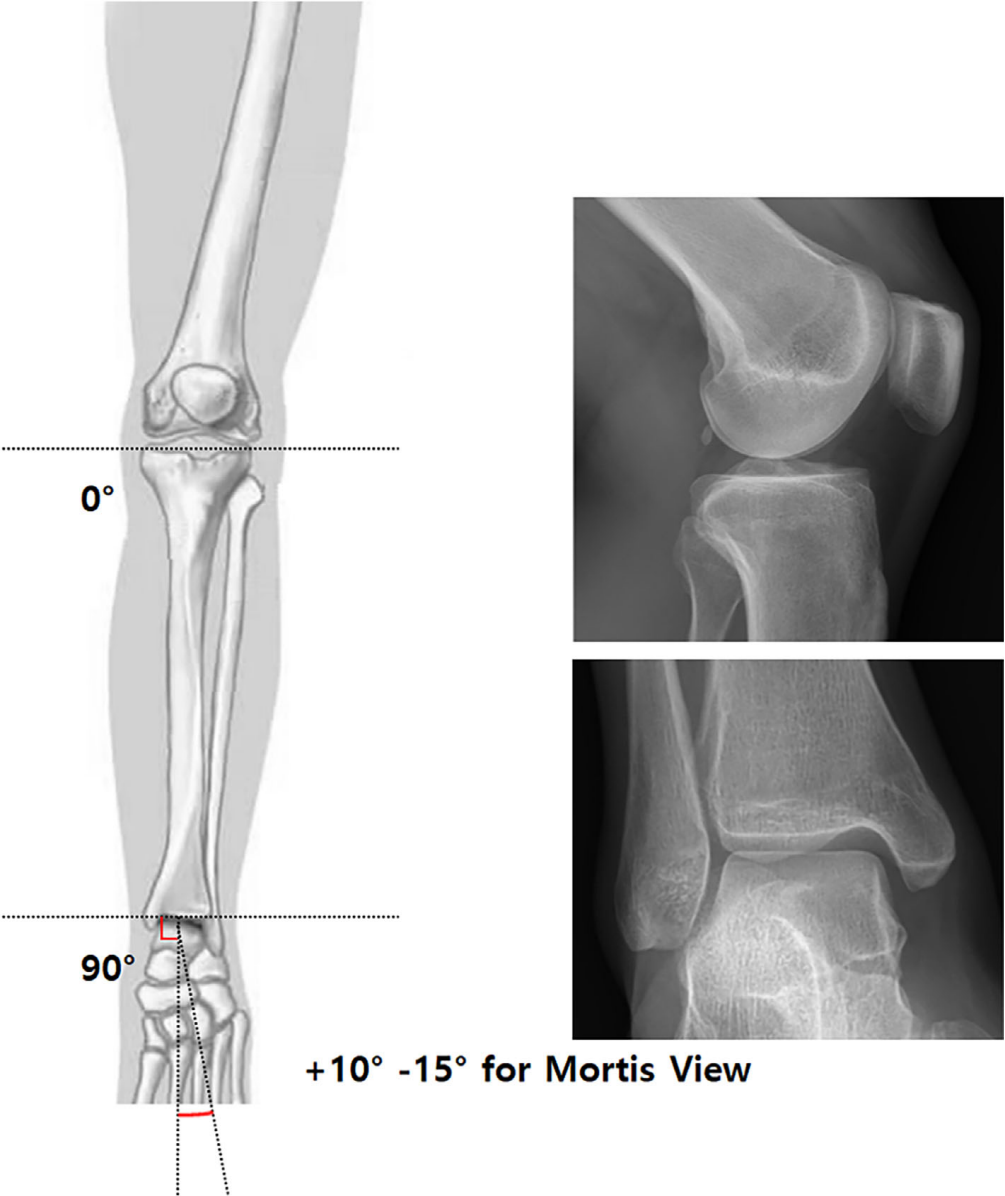
Clementz technique is performed to measure the tibial rotation under C‐arm image intensifier by measuring two reference point on patient's knee and ankle

Because preoperative CT was not available for all patients, tibia rotation was measured using the Clementz measurement technique in the operating room^17^. The patient lay supine with a fully extended knee and the leg was turned around its longitudinal axis so that the posterior contours of the femoral condyles in the lateral view were superimposed on each other in the horizontal plane. The fluoroscope was then moved to the level of the ankle, and in the transverse plane, the C‐arm was rotated until a tangential image of the inner surface of the medial malleolus was obtained; the distal line of the reference was then established (Fig. 2).

All radiological parameters were compared with the parameters from the contralateral side, and preoperative and postoperative radiologic parameters were compared between the SP and IMN groups. Further, the relationships of ankle alignment changes and hindfoot alignment changes were evaluated between local radiologic parameters and hindfoot alignment.

### Statistical Analysis

Descriptive statistics are presented as the mean and standard deviation, the median and interquartile range, or the number and percentage, as appropriate. Groups were compared using the *t*‐test, Mann–Whitney *U*‐test, or Fisher exact test, as appropriate. Moreover, we evaluated correlations with Pearson's correlation analysis. Logistic regression analysis was used to model the effects of radiographic parameters of hindfoot alignment caused by alignment changes after surgery. We chose a significance cutoff of *p* < 0.05 for two‐sided tests.

For the interobserver reliability, all radiographic parameters except for the tibial rotation were measured independently by the two authors, each with 5 years of experience as an orthopaedic surgeon. In case of tibial rotation, since it has been measured in the operation room, it was measured twice by a single surgeon. Before the independent measurement, the radiographic data for 10 patients were evaluated in cooperation by the two observers to ensure consistency in the measurement approach. To assess intraobserver reliability, all measurements were re‐evaluated by the same observer at 3 weeks and 2 months after the initial measurements. The interobserver and intraobserver reliabilities were expressed in terms of the intraclass correlation coefficient (ICC), which was classified as follows: >0.75, excellent; 0.4–0.75, fair to good; and <0.4, poor. The ICCs for interobserver and intraobserver reliability are listed in Table 1. LDTA, TTA, talar tilt angle, and all hindfoot parameters showed satisfactory results, except for tibia rotation angle. We did all statistical analyses with the SPSS version.

**TABLE 1:** Interobserver and intraobserver reliability of radiologic parameters of ankle, tibia, and hindfoot

Measurement	Intraobserver reliability		Interobserver reliability	
	ICC	95% *CI*	ICC	95% *CI*
LDTA (former TAS)	0.90	0.79–0.97	0.92	0.87–0.99
TTA (tibiotalar angle)	0.91	0.79–0.97	0.90	0.79–0.96
Talar tilt angle	0.88	0.78–0.97	0.89	0.78–0.98
Tibial rotation angle	0.71	0.52–0.90	Not applicable	
Hindfoot alignment angle	0.92	0.87–0.97	0.93	0.86–0.99
Hindfoot moment arm	0.83	0.75–0.91	0.84	0.76–0.92
Calcaneal pitch	0.87	0.74–0.97	0.88	0.76–0.97
Lateral Meary angle	0.86	0.75–0.93	0.89	0.78–0.98

Abbreviations: CI, confidence interval; ICC, intraclass correlation coefficient.

## Results

We studied 92 patients with distal tibia fractures treated with IMN (39 patients) and SP (53 patients); 37 patients in the IMN group and 47 in the SP group met the inclusion criteria. The average follow‐up period was 19 ± 5.7 months from the surgery in both groups. All patients ultimately healed, with an average of 26 weeks for complete union in both groups. Complications were similar between the two groups.

### Clinical Comparison between SP and IMN

The clinical characteristics of SP and IMN are as follows. The mean VAS score was 2.7 ± 1.3 in the IMN group and 3.0 ± 1.5 in the SP group (*P* = 0.121). The mean AOFAS score was 88.1 ± 6.8 in the IMN group and 87.3 ± 5.7 in the SP group (*P* = 0.519). The AOFAS and VAS scores were not significantly different between the two groups.

### Radiologic Comparison between SP and IMN

We compared the radiological parameters of the operated and contralateral ankles and feet (Table 2). Because we could not measure the preoperative condition, we assumed that the parameters of the contralateral side were in the preoperative state. Changes in hindfoot alignment angle, calcaneal pitch, and Meary angle showed no significant differences between the groups. However, the hindfoot moment arm was slightly increased valgus in the IMN group. There were no significant differences in radiologic parameters between the two groups.

**TABLE 2:** Radiologic parameter comparison

Radiologic parameter	IMN group (37)		SP group (47)		*P* value
	Operated limb	Contralateral limb	Operated limb	Contralateral limb	
Fracture angle_ AP	1.3 ± 2.1	‐	1.1 ± 1.9	‐	0.17
Fracture angle _Lat	2.8 ± 5.9	‐	2.4 ± 3.9	‐	0.67
Tibial rotation angle	3.4 ± 5.9	‐	2.7 ± 4.8	‐	0.28
Radiologic parameters of ankle and tibia					
LDTA (former TAS)	86.1 ± 2.9	87.3 ± 2.4	86.5 ± 3.7	87.5 ± 2.3	0.57
TTA (tibiotalar angle)	2.1 ± 0.4	1.5 ± 0.3	1.8 ± 0.3	1.3 ± 0.3	0.73
Talar tilt angle	1.3 ± 0.5	1.2 ± 0.3	1.8 ± 1.2	1.2 ± 0.4	0.28
Radiologic parameters of hindfoot					
Hindfoot alignment angle	6.8 ± 4.8	6.2 ± 3.5	5.9 ± 5.6	5.9 ± 3.7	0.16
Hindfoot moment arm	4.6 ± 5.9	3.5 ± 4.8	3.2 ± 4.2	3.1 ± 4.3	0.08
Calcaneal pitch	23.1 ± 6.5	21.2 ± 5.3	23.8 ± 5.6	23.4 ± 5.1	0.23
Lateral Meary angle	8.9 ± 7.8	7.8 ± 5.8	9.8 ± 6.8	9.2 ± 5.4	0.51

### Relationship between Ankle Alignment Changes and Hindfoot Alignment Changes

There was a significant low correlation between angulation at the fracture site in the coronal view and hindfoot alignment angle (*R* = 0.37), hindfoot moment arm (*R* = 0.38). A significant high correlation was noted between transverse tibial rotation angle and hindfoot alignment angle (*R* = 0.71), hindfoot moment arm (R = 0.79). Only transverse rotation angle had a strong correlation (*P* < 0.05) with hindfoot alignment changes on regression analysis while coronal fracture angle was not statistically significant.

## Discussion

### Treatment Options for the Surgical Management of Distal Tibial Fracture

AO/ASIF type 42‐A1 and A2 fracture of the distal tibia is considered to be a simple fracture. According to the AO principles of fracture management, a simple fracture needs anatomical reduction, rigid fixation, absolute stability, and primary bone healing. The traditional technique of open anatomic reduction and internal fixation of the distal tibia fractures requires extensive soft‐tissue dissection and often leads to subsequent periosteal injury. Accordingly, high rates of complications, including postoperative infection, delayed union, and nonunion, have been reported^5^, ^10^. As an alternative, IMN, without exposure of the fracture site, has been considered a good solution to these problems^4^, ^6^, reducing both iatrogenic soft‐tissue injury and damage to the blood supply.

### Various Outcomes of SP and IMN

Recently, there have been various reports on the clinical and radiological results of AO‐OTA 43‐A1 and 43 A2 distal tibia metaphyseal fractures after treatment with SP and IMN. Generally, no differences have been detected for bone‐union rates, wound complications, and superficial or deep infections between SP and IMN. However, IMN resulted in shortened time to union and less bleeding during surgery compared with SP, whereas the incidence of malalignment and anterior knee pain is less reported with SP^18^.

In the previous study of Falzarano *et al*.^19^, there was no significant difference in clinical outcome according to implant type while complication rate is higher in IMN group. In this report, accurate reduction in tibial fracture is the most important factor in future outcome.

Nevertheless, Janssen *et al*.^16^ reported that there was no difference in the incidence of malalignment between the two surgical methods, perhaps because of various factors, such as the use of block screws, screw fixation after proper reduction, and improvement of the IMN device.

Reduction and stable fixation of distal extra‐articular tibia fractures with IMN is often technically challenging, because of a large medullary cavity within a short distal fragment. In order to solve such a problem, novel nail designs and surgical techniques have been described during the last two decades, such as multi‐directional and angle‐stabilized distal locking systems and locking screw holes at the tips of nails, and use of (poller) blocking screws to narrow the medullary cavity^20^, ^21^.

In this study, we reviewed not only the previous reports of clinical outcomes, alignment complication, and knee pain, but also the foot and ankle alignment and foot functional score. No previous study has investigated foot and ankle alignment and foot functional scores after distal tibia diaphyseal fracture. Unlike our predictions, there was no difference in foot and ankle alignment and foot functional score between the SP and IMN groups.

### Factors Contributing to Hindfoot Alignment in Distal Tibial Fracture

**Fig. 3:**
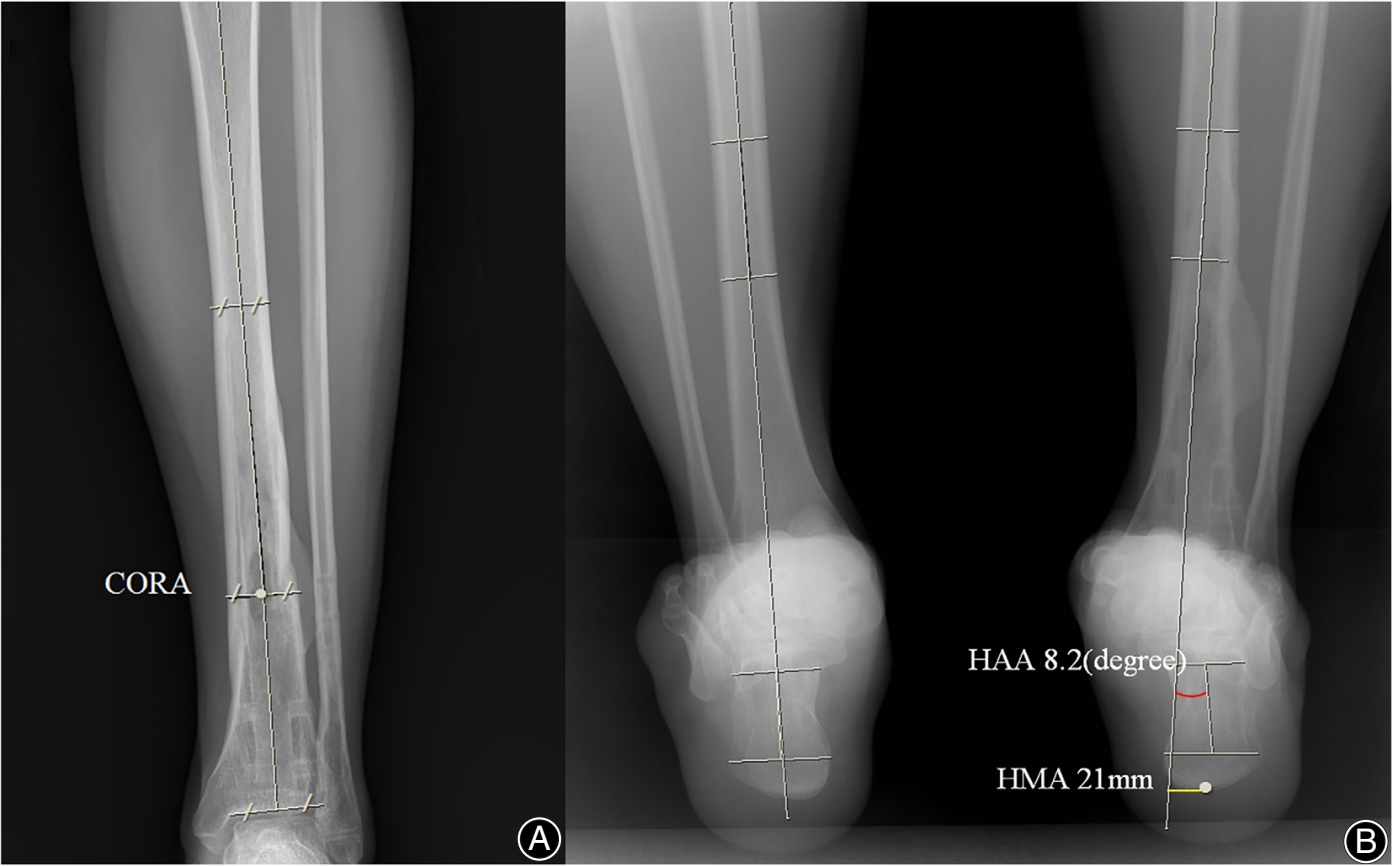
30‐year‐old man with distal tibia fracture had fracture union. There was minimal valgus deformity on cornal axis less than 0.5′ however had 5.2′ external transverse axis rotation. Radiographs showing 8.2′ valgus deformity on hindfoot alignment angle (HAA) and 21‐mm hindfoot moment arm (HMA) translation on hind foot alignment

In terms of the relationship between the change in the fracture site and hindfoot alignment, transverse rotation was significantly related to the hindfoot alignment in both the SP and IMN groups. On the other hand, coronal plane angulation was not significantly related to hindfoot alignment (Fig. 3), and these findings are difficult to understand intuitively. Wang *et al*. reported that compensation for coronal plane alignment occurs at the subtalar joint^22^. Knupp *et al*. also reported the compensation of the subtalar joint^23^. The absence of a relationship between coronal plane angulation and hindfoot alignment in our data probably resulted from such subtalar compensation.

### Factors Contributing to Hindfoot Alignment in Distal Tibial Fracture

There were limitations to this study. First, it dealt with only a few patients and had different numbers of them in the SP and IMN groups. Nevertheless, all patients with distal tibia metaphyseal fractures treated with SP or IMN had full‐term follow‐up. In the follow‐up periods, various radiologic parameters were measured for all patients, including hindfoot alignment. Second, the ischemic injury after fracture or trauma in the lower leg induces shortening or contracture of muscles that causes ankle joint stiffness or toe deformation. Rarely, it can also cause foot and toe deformations, such as checkrein deformity, clawing toe deformity, lesser toe deformities, and cavus foot^24^, ^25^. Furthermore, alterations of hindfoot alignment may also take place. However, these changes were considered difficult to quantify and were not measured in this study. In order to minimize changes in the result, the effect of hindfoot alignment by soft tissue was reduced by the exclusion of AO classification 42‐A3. Last, since we were not able to obtain preoperative CT measurements from all the patients, the degree of transverse rotation had to be measured by the Clementz technique intraoperatively, which showed insufficient reliability.

Nevertheless, this study demonstrated that postoperative alignment changes in distal tibial fractures may affect hindfoot alignment, although some angular malunion in the coronal plane could be compensated for in the subtalar joint. In future research, a multi‐center study to analyze a large cohort would be necessary to clarify these issues.

## Conclusion

In this study, similar union and complication rates were noted when treating non‐articular metaphyseal distal tibia fractures with SP compared with IMN. Angulation in the coronal plane after internal fixation may have a minimal effect on the change in hindfoot alignment because of the compensation in the subtalar joint. However, rotation in the transverse plane markedly influenced change in hindfoot alignment. Therefore, regardless of the fixation method, patients treated for distal tibia fracture require close attention for changes in hindfoot alignment because of transverse rotation during surgery.
